# Psychiatric Morbidity Among Youth Patients at Nakuru County Referral and Teaching Hospital in Kenya: A Cross-Sectional Study

**DOI:** 10.5964/ejop.14511

**Published:** 2025-08-29

**Authors:** Mercy Cheptoo Kipkemboi, Teresa Ndilu Mutavi, John Maina Mburu

**Affiliations:** 1 Department of Psychiatry, University of Nairobi, Nairobi, Kenya; 2 Clinical Psychology Department, HealthX Africa, Nairobi, Kenya; Stanford University, Stanford, CA, USA

**Keywords:** psychiatric morbidity, youth, youth patients, 18 – 35 years, Kenya

## Abstract

There is a decreased life span of 10 – 15 years in persons with psychiatric illnesses in contrast with the public population; hence interventions on first onset may improve some end results. This article explores the psychiatric morbidity among youth patients of age group 18 – 35 years. The study implemented a cross-sectional descriptive design. The study was carried out at Nakuru County Referral and Teaching Hospital in Kenya: 385 youth patients were sampled for this study using simple random sampling. There were more female (55.3%) participants compared to male (44.7%). Marital status and employment status were found to have a statistically significant association with psychiatric morbidity. The singles (*p* = .024) had an OR (4.771) higher chance of having a psychiatric morbidity as compared to the married. On the other hand, those who were widow/widower (*p* = .016) had an OR (5.650) times of developing a mental illness compared to the married. In conclusion, the prevalence of psychiatric morbidity among youth outpatients of the age bracket 18 – 35 years at Nakuru County Referral and Teaching Hospital stands at 46.5%. Marital status and employment status were noted to have a statistically significant link with psychiatric morbidity. Regular psychological assessments should be conducted as part of treatment evaluations for patients to get other more interventions necessary for them, bettering their health outcome broadly.

Among the most rampant leading causes of global burden of disease are psychiatric disorders (Meyer & Ndetei, 2016). Mental illnesses have risen to be a major public health challenge (Wu et al., 2023). In addition, the World Health Organization indicates that roughly 1 billion individuals struggle with psychiatric disorders (Wu et al., 2023). Psychiatric illness is a disorder manifested by medically significant distress in a person’s behavior, emotions and cognitive functions which indicates a disorder in the biological, developmental and psychological activities of psychiatric functions (DSM-5, 2013; Tse & Haslam, 2023). The frequency of common psychiatric disorders in Kenya accounts for 10.3% (Ministry of Health of Kenya, 2021). However, Kenya is considered a youthful country since most of its inhabitants constitutes of age bracket 18 – 34 years (Chumo, 2018). A youth is a person who falls in the age category of 18 – 35 years old as per the 2010 constitution of Kenya (Hope, 2012) being used presently. This age group has significant social and economic chances but correspondingly extensive challenges (Ministry of ICT, Innovation and Youth Affairs of Kenya, 2019). However, if consistent education and quality healthcare are provided, the nation will experience advantages from this group (Mwadime, 2022). Therefore, there is need to understand the specific psychiatric morbidity among the youths since high rates may affect their productivity in this crucial stage. Previous studies show that the general practitioners in hospitals may lack the expertise necessary to perform psychiatric assessments; hence they rarely provide these critical evaluations (Zhang et al., 2019). Some patients seeking general treatment in hospitals may have undiagnosed psychiatric illnesses which may endanger their lives. For instance, persons with depressive symptoms have high risk of taking their own life (World Health Organization, 2022). The results of this study will inform policy makers in enhancing policies that will strengthen the psychological mental health of youths aged 18 – 35 years in response to psychiatric morbidity rate in Nakuru County Teaching and Referral hospital and other similar hospitals. It will also give insight to youths who are not aware of their mental health status; to take caution and seek the necessary help for those in need. The primary objective of this study is to determine the psychiatric morbidity among youth patients aged 18 – 35 years and identify the associated demographic factors, to inform effective public health interventions.

## Method

The target population was youths aged 18 – 35 years being seen at the outpatient casualty in Kenya, for general medical conditions by general medical doctors. A simple random sampling technique was used to randomly select participants. A complete list of the registered outpatients was acquired at the outpatient’s casualty area in the hospital. A list of randomly generated numbers using excel was done, for each participant. A lottery method was used; participants were assigned a number, which were randomly picked until required sample size of 385 participants was achieved. The sample size was found using Cochran’s (1977) formula. The Mini International Neuropsychiatric Interview (M.I.N.I) tool Version 7.0.0 was administered together with a socio-demographic questionnaire. For participants to complete the interview, it approximately took 20 – 30 minutes. The socio-demographic data collection sheet included interviewer identification, location, age group (18 – 23, 24 – 29 and 30 – 35), gender, marital status, education level, employment status and religion. The MINI Instrument is a short organized diagnosis assessment tool whose author is Sheehan (2015), and the interview contained 17 disorders recognized as modules. Psychiatric morbidity occurs when a participant fits into one of the categories of disorders included in the MINI instrument, whose questions are derived from the Diagnostic and Statistical Manual of Mental Disorders V (DSM-5) (Kwobah et al., 2017).

## Results

Participants included in the present study were 385: 55.3% of the participants were female while 44.7% were male. The majority of the participants were 24 – 29 years old (39.2%) followed by age group 18 – 23 (31.7%) and age group 30 – 35 years (29.1%). Most participants were single (53.5%), married (41.6%), widow/widower (1.3%), and divorced/separated (3.6%). Most participants (38.7%) had completed a secondary level of education, while 22.3% had not completed tertiary, 16.4% had completed tertiary, 11.4% had not competed secondary, 7.8% had completed primary, 2.6% had not completed and 0.8% had no level of education. Almost half of the participants (45.5%) were unemployed, while self-employed comprised 35.8% and less than a quarter of the participants (18.7%) were employed. Majority of participants (91.9%) were Christians, 5.2% of Muslims, 1.6% belonged to other faiths and 1.3% identified as atheists. (Table 1)

**Table 1:** Socio Demographic Profiles of Study Participants

Socio-demographic profile	Category	Frequency	Percentage
Marital status	Single	206	53.5
	Married	160	41.6
	Widow/widower	5	1.3
	Divorced/separated	14	3.6
	Total	385	100
Education level	None	3	0.8
	Primary not completed	10	2.6
	Primary completed	30	7.8
	Secondary not completed	44	11.4
	Secondary completed	149	38.7
	Tertiary not completed	86	22.3
	Tertiary completed	63	16.4
	Total	385	100
Employment status	Employed	72	18.7
	Self-employed	138	35.8
	Unemployed	175	45.5
	Total	385	100
Religion	Christian	354	91.9
	Muslim	20	5.2
	Atheist	5	1.3
	Others	6	1.6
	Total	385	100

The prevalence of psychiatric morbidity among youth patients in the hospital was 46.5%. The psychiatric illness with the highest prevalence was major depressive episode (MDE) (21.8%) systematically followed by; suicidality (8.8%), alcohol use disorder (AUD) (8.6%), suicidal behavior disorder (8.3%), post-traumatic stress disorder (PTSD) (6.2%), generalized anxiety disorder (GAD) (5.2%), social anxiety disorder (SAD) (4.9%), panic disorder (2.9%), manic and hypomanic episodes (2.3%), agoraphobia (2.3%), obsessive-compulsive disorder (OCD) (2.3%), substance use disorder-non-alcohol (SUD-NA) (2.1%), psychotic disorder lifetime (1.8%), antisocial personality disorder (ASPD) (1%), mood disorders with psychotic features lifetime (0.8%), binge eating disorder (0.8%), psychotic disorders current (0.5%), anorexia nervosa (0.5%), mood disorders with psychotic features current (0.5%) and bulimia nervosa (0.3%) (Table 2).

**Table 2:** Prevalence of Psychiatric Morbidity Among Youth Patients in the Hospital

Variable	Frequency	Percentage
Major Depressive Disorder	84	21.8
Suicidality	34	8.8
Alcohol use disorder	33	8.6
Suicidal behavior disorder	32	8.3
Post-traumatic stress disorder	24	6.2
Generalized anxiety disorder	20	5.2
Social anxiety disorder	19	4.9
Panic disorder	12	3.1
Manic and hypomanic episodes	9	2.3
Agoraphobia	9	2.3
Obsessive compulsive disorder	9	2.3
Substance use disorder (non-alcohol)	8	2.1
Psychotic disorders lifetime	7	1.8
Antisocial personality disorder	4	1
Mood disorder with psychotic features lifetime	3	0.8
Binge eating	3	0.8
Psychotic disorders current	2	0.5
Mood disorders with psychotic features current	2	0.5
Anorexia nervosa	2	0.5
Bulimia nervosa	1	0.3

Marital status and employment status were found to have a statistical relationship with psychiatric morbidity. Participants who identified as single had an odds ratio (OR) higher chance of having a psychiatric morbidity as compared to the married **(***p* = .024), OR 4.771. Participants who were a widow/widower had an OR 5.650 times of developing a mental illness compared to the married. Those who were divorced/separated had a low odds ratio (0.000) of having a psychiatric morbidity compared to those who were married **(***p* = .016). Participants who were self-employed indicated strong odds of developing a psychiatric morbidity as compared to those who were unemployed (*p* = .007, OR 2.440); **(***p* = .018).

Logistic regression analysis indicated age and marital status having a strong significant statistic link with MDE. Despite age being significant to MDE, the MDE rates in both the 24 – 29 and 30 – 35 year old age groups were lower than those in the 18 – 23 year old age group (*p* = .009) (OR: 0.323); (*p* = .024) (OR: 0.370).

Most of the participants had met a single diagnosis criterion by 26.8% (103), 9.9% (38) had 2 comorbidities, 6.8% (26) had 3 comorbidities while 3.1% (12) had 4 comorbidities (Table 3). 

**Table 3:** Psychiatric Comorbidities Among Youth Patients

Number of comorbidities	Frequency	Percentage
0	206	53.5
1	103	26.8
2	38	9.9
3	26	6.8
4	12	3.1
Total	385	100

## Discussion

The present study was investigating the associations between psychiatric morbidity and being a youth. The outcome indicated that majority of the participants were women. This could be because health care-seeking tendencies are determined by various personal traits such as age and gender, whereby men are inadequately represented in medical care, and it is hard to comprehend why because it’s a continuing matter (Thompson et al., 2016). Moreover, the male gender was also noted to inadequately utilize the medical services specifically health screening and primary care (Mursa et al., 2022) and to also use medical services minimally as compared to the female gender (Australian Institute of Health and Welfare, 2019; Mursa et al., 2022). Nevertheless, more youthful patients both men and women are ready to get health care assistance as opposed to older patients (Thompson et al., 2016) as also seen in our findings.

This study indicates that the most prevalent mental illness in youth patients of age bracket 18 – 35 years was major depressive episode (21.8%) this was less in contrast to research done by Greenberg et al. (2021) that showed major depressive disorder to be at 53.7%% among those in age bracket of 18 – 34. However, age category 30 – 35 years had the highest level of major depressive episode suggesting that there is an increase of major depressive episode with increase in age. Similarly, Solmi et al. (2022) noted that average age onset of depressive disorder was 30 – 35 years. This was in contrast with Villarroel and Terlizzi (2020), who noted that those who were of age bracket of 18 – 29 years (21.0%) had the majority incidences of symptoms of depression while those in age group 30 – 44 years (16.8%) had the least symptoms.

The level of education indicated a correlation to major depressive episode; it was noted that those who were of secondary level and below had higher prevalence of major depressive episode compared to those of tertiary level and above. This was in alignment with the findings of Lee et al. (2016). In addition to that, Arias-de la Torre et al., (2021) also noted in their study that education level impacts outcome of MDD.

In this study divorced/separated respondents (64.3%) showed a high level of major depressive episode this was in alignment with Gutiérrez-Rojas et al. (2020), whose results indicated a strong correlation between major depressive disorder and being divorced/separated. Additionally, Bulloch et al.’s (2009) findings concluded that individuals who are divorced or separated have higher risk of developing major depressive disorder. Married participants (24.4%) followed in second position. This was in contrast to the findings of Islam and Adnan (2017), which indicated that married individuals 56% had a tendency to develop depression the highest.

Those who were unemployed (82.4%) had a slightly higher percentage of major depressive episode as opposed to those who were employed (81.0%) and self-employed (81.4%). This could be because unemployment is persistently linked with increased rates of depression in young adults (McGee & Thompson, 2015). This finding is also consistent with findings on depression and unemployment in young adults by McGee and Thompson (2015).

AUD had a prevalence rate of 8.6% and indicating a correlation with gender, education level and employment status. The male gender (14.0%) used more alcohol as compared to the female (4.2%), this could be because males and females are attributed with contrasting societal morals and norms (Gilbert et al., 2019; Goh et al., 2024). Similarly, Maxwell et al. (2022) and Goh et al. (2024) supported that sociocultural viewpoints, customary masculine norms particularly could be the reason for men’s high levels of alcohol consumption with possibility of having more alcohol related challenges. Those who were of secondary level of education and below indicated a higher intake of alcohol use as compared to those of tertiary level and above, this corresponded with Murakami and Hashimoto’s (2019) findings which indicated that less educational level was correlated with more risk of having a drinking challenge. Similarly, Norström and Landberg’s (2020) findings also supported this. Moreover, lesser educational level predisposes one to being a binge drinker due to occasional predisposition to social distress and poor health enlightenment about dangers of alcohol consumption (Murakami & Hashimoto, 2019).

Employment status was also linked to alcohol use disorder whereby the self-employed had 15.2% AUD while those employed had 11.1% and those unemployed had 2.4% this was similar to Kiarie (2021), who found a significant link between self-employment and alcohol use. However, this contrasted with Collins’s (2016) findings, which indicated that unemployment was what was found to be greatly linked with heavy alcohol consumption and alcohol use disorder. Although age was not statistically significant to AUD, the age group of 30 – 35 rated a higher percentage (11.6%) of AUD in contrast to other age groups which was consistent with World Health Organization (2022), and Wysokińska and Kolota (2022), who noted those of age group 25 – 45 years had a peak in alcohol usage. This differentiated with Delker et al.’s (2016) findings which indicated a heightening of alcohol use in age group 18 – 29 years.

Marital status and employment status were noted to have a statistically significant link with psychiatric morbidity. This was in contrast with Kwobah et al. (2017) who noted that there was no link found between marital status and employment status having an association with psychiatric morbidity. On the other hand, Bulloch et al. (2009) indicated an association of marital status and psychiatric illness while Eid et al. (2013) indicated an association between employment status and psychiatric illness.

### Limitations and Future Studies

The main limitation noted was that some youth patients would get tired while answering questions due to a long questionnaire which had long different questions for 16 different mental disorders, hence future studies would benefit from using a brief tool with just as good validity as Mini International Neuropsychiatric Interview Version 7.0.0. To tackle this limitation, the participants who did not wish to continue with the interview were allowed to leave since participation was voluntary.

This research was a cross-sectional study whose duration was 3 months hence a longitudinal study may be of importance to further evaluate the temporality of the results and predictors of psychiatric morbidity among youth patients (18 – 35 years). The present study found that self-employment was significantly correlated with psychiatric morbidity. Future studies may benefit looking into the specific factors related to self-employment that may play a role in the vulnerability of psychiatric morbidity in the age 18 – 35 years.

### Conclusion

The primary outcome of this research indicates that there is a correlation between psychiatric morbidity and being a youth. Major depressive disorder and alcohol use disorder had the highest prevalence among the youths. MDD indicated a relationship with increase in age while marital status and employment suggested a significant relationship with psychiatric morbidity. These findings could help inform policy makers in enhancing policies that strengthen the mental health of youths of 18 – 35 years.

## Supplementary Materials

**Table:** 

Type of supplementary materials	Availability/Access
Code	
SPSS Syntax.	Kipkemboi (2025)
Material	
a. Interview protoccol in Kiswahili and English.	Kipkemboi (2025)
b. MINI 7.0.0 study material in English.	Kipkemboi (2025)
c. MINI 7.0.0 study material in Kiswahili.	Kipkemboi (2025)
Other	
Codebook/Data dictionary.	Kipkemboi (2025)

## Data Availability

Data can be accessed through the Open Science Framework. See Kipkemboi (2025).
